# New Chloramphenicol Derivatives from the Viewpoint of Anticancer and Antimicrobial Activity

**DOI:** 10.3390/antibiotics8010009

**Published:** 2019-01-29

**Authors:** Panagiota C. Giannopoulou, Dionissia A. Missiri, Georgia G. Kournoutou, Eleni Sazakli, Georgios E. Papadopoulos, Dionissios Papaioannou, George P. Dinos, Constantinos M. Athanassopoulos, Dimitrios L. Kalpaxis

**Affiliations:** 1 Department of Biochemistry, School of Medicine, University of Patras, GR-26504 Patras, Greece; p.giannopoulou@upnet.gr (P.C.G.); gkurnutu@upatras.gr (G.G.K.); dim.kalpax@gmail.com (D.L.K.); 2 Laboratory of Synthetic Organic Chemistry, Department of Chemistry, University of Patras, GR-26504 Patras, Greece; dionysia.misiri@gmail.com (D.A.M.); dapapaio@upatras.gr (D.P.); 3 Laboratory of Public Health, School of Medicine, University of Patras, 26504 Patras, Greece; esazakli@med.upatras.gr; 4 Department of Biochemistry & Biotechnology, University of Thessaly, Biopolis, GR-41500 Larissa, Greece; geopap@uth.gr

**Keywords:** Chloramphenicol, polyamines, conjugates, mitochondrial ribosome, molecular dynamics simulations, antimicrobial activity, anticancer activity, protein biosynthesis, antibiotics

## Abstract

Over the last years, we have been focused on chloramphenicol conjugates that combine in their structure chloramphenicol base with natural polyamines, spermine, spermidine and putrescine, and their modifications. Conjugate **3**, with spermidine (SPD) as a natural polyamine linked to chloramphenicol base, showed the best antibacterial and anticancer properties. Using **3** as a prototype, we here explored the influence of the antibacterial and anticancer activity of additional benzyl groups on N1 amino moiety together with modifications of the alkyl length of the aminobutyl fragment of SPD. Our data demonstrate that the novel modifications did not further improve the antibacterial activity of the prototype. However, one of the novel conjugates (**4**) showed anticancer activity without affecting bacterial growth, thus emerging as a promising anticancer agent, with no adverse effects on bacterial microflora when taken orally.

## 1. Introduction

Chloramphenicol (CAM) is an effective antibiotic with bacteriostatic action against Gram-positive and Gram-negative bacteria and was the first broad-spectrum antibiotic to be used clinically. However, the undesired toxicity resulting from CAM administration restricts its clinical use. Case reports indicate that CAM is responsible for hematological disorders such as bone marrow depression and aplastic anemia. While the first is a rare complication, the second is more frequent and was ascribed as a mitochondrial protein synthesis disorder [1,2,3,4]. Despite its side effects, CAM remains one of the best-studied protein synthesis inhibitors, and efforts towards its derivatization have continued in order to provide a new generation of compounds with better antibacterial activity and minimal side effects [5]. Numerous functional and structural studies have concluded that CAM binds to the large ribosomal subunit occupying the A-site of the peptidyltransferase center (PTC) [6,7,8]. Its binding allows the delivery and initial binding of the A-site aminoacyl-tRNA but prevents its full accommodation at the PTC, which brings the newly incoming aa-tRNA close to the P-site peptidyl-tRNA. Additionally, CAM, like other peptidyltransferase (PTase) inhibitors, is not a general protein synthesis inhibitor but a context-specific one [5,9]. Over the last years, we have been focused on CAM derivatization involving the conjugation of the CAM-base with natural polyamines (PAs), in an attempt to develop new effective derivatives which avoid the undesired side effects of CAM [10,11]. Our continuous efforts aim to combine two different main and contrary players of protein synthesis on the same molecule.

PAs are low molecular weight aliphatic amines which are associated with multiple phenomena within cells and specifically with ribosome function and protein biosynthesis [12,13]. Their significance for growth and survival is proven by the fact that the biogenic PAs putrescine (PUT), spermidine (SPD) and spermine (SPM) are found in almost all living organisms. Bacteria and mammalian cells are able to synthesize de novo PAs which they need, but also express polyamine transporters (PATs) that mediate the transport of extracellular PAs [14,15]. Additionally, cancer cells which are characterized by a pronounced proliferation rate need higher levels of metabolites including PAs. Indeed, many studies have confirmed the existence of higher levels of PAs in cancer cells compared to normal, derived from both elevated biosynthesis and transporter expression on the cell membranes [16]. Thus, PA metabolism and transport inhibition appear as promising targets for the treatment of neoplasms [17].

We have recently reported the synthesis of a series of conjugates (PA–CAM conjugates) of the naturally occurring PAs—PUT, SPD and SPM, with CAM—which showed comparable binding affinities to CAM for the 70S *E. coli* ribosome [10]. Among them, conjugate **3** was the most potent inhibitor of PTase activity (*K_i_*: 0.98 µM) and also the most potent antibacterial agent, inhibiting the growth of different microbial cells with comparable (wild-type *E. coli* strains) potency to CAM, or even double the potency (mutant *E. coli* strains) [10]. Furthermore, conjugate **3** showed superior selectivity and activity than CAM against human mesothelioma ZL34 and immortalized human mesothelial Met5A cells [10]. These improved activities of the conjugate **3** were attributed to the introduction of two benzyl moieties on the N8 amino group of the SPD fragment linked to CAM, which resulted in an increased lipophilicity of the molecule, which could facilitate its passage through the cell membrane. However, despite the mentioned advantages of **3** compared with the rest of the PA–CAM conjugates, it was not superior to CAM in inhibiting wild-type *E. coli* strains, indicating that cellular permeability remained a significant barrier for the use of the compound in the treatment of bacterial infections.

**Figure 1:**
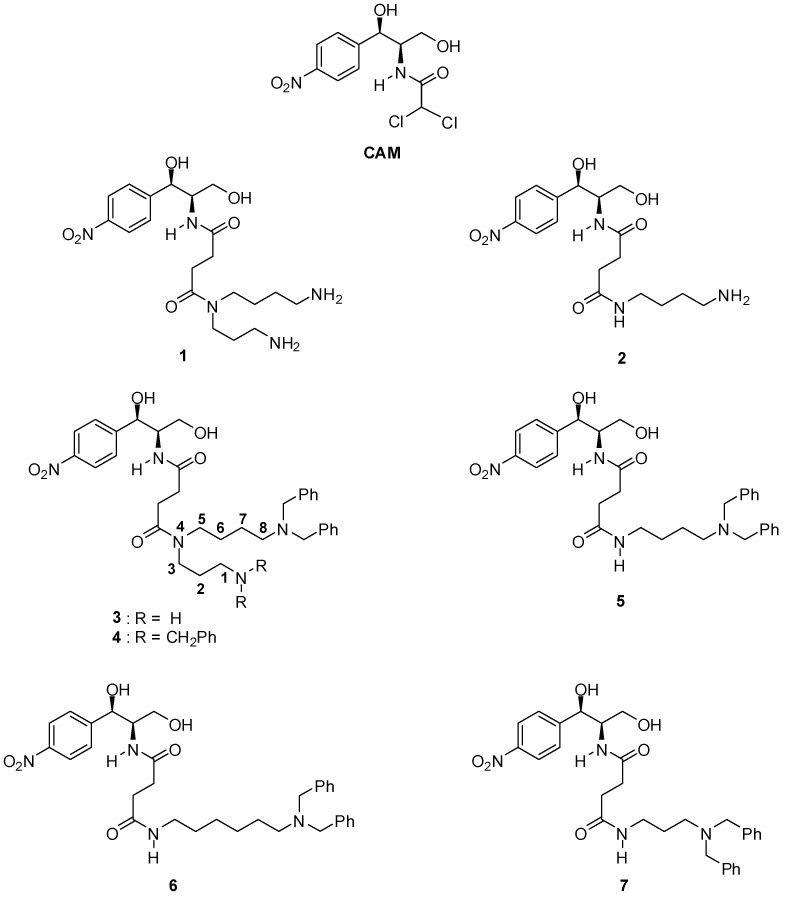
Structures of compounds encountered in the present work.

Taking conjugate **3** as a prototype, we present now the synthesis and the evaluation of the antimicrobial and antitumor activity of new conjugates, which were designed in such a way to allow conclusions regarding the effect of (a) introducing additional benzyl moieties on the N1 of the SPD skeleton, (b) deleting the aminopropyl moiety of the SPD skeleton, and (c) extending or shortening the aminobutyl moiety on their biological activity. More precisely, we designed and synthesized the four new conjugates **4**–**7** (Figure 1). In conjugate **4**, two additional benzyl groups replaced the hydrogen atoms at the N1 position of the SPD moiety, whereas in conjugate **5** the aminopropyl moiety was omitted. Conjugates **6** and **7** constitute analogs of conjugate **5** in which the aliphatic chain of the aminobutyl moiety was either extended or shortened. 

## 2. Materials and Methods

### 2.1. Synthesis of PA–CAM Conjugates

**Scheme 1:**
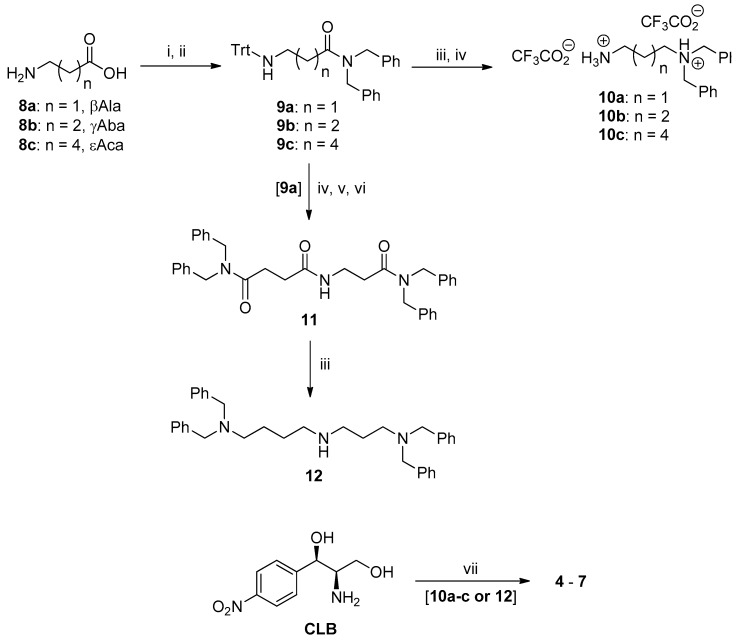
Synthesis of polyamine (PA)–chloramphenicol (CAM) conjugates **4**–**7**. Reagents and reaction conditions: (i) (a) Me_3_SiCl, DCM/MeCN (5:1), reflux, (b) TrtCl/Et_3_N, (c) MeOH, 85–97%; (ii) (PhCH_2_) _2_NH, *O*-(benzotriazol-1-yl)-*N*,*N*,*N*′,*N*′-tetramethyluronium hexafluorophosphate (HBTU), Et_3_N, 65–80%; (iii) lithium aluminium hydride (LAH), THF, reflux, 60–80%; (iv) trifluoroacetic acid (TFA)/CH_2_Cl_2_(1:9 or 2:8 *v*/*v*), Et_3_SiH, 85–95%; (v) succinic anhydride, DIEA, DMF; (vi) (PhCH_2_)NH, HBTU, ethyldiisopropylamine (DIEA), 90% over the two steps; (vii) (a) succinic anhydride, DMF, (b) **10a-c** or **12**, HBTU, DIEA, DMF, 60–73%.

The synthesis of the new PA–CAM conjugates **4–7** is depicted in Scheme 1. It involves the one-pot acylation of the commercially available chloramphenicol base (CLB) with succinic anhydride followed by coupling with the appropriate *N*^1^,*N*^1^-dibenzylated linear diamines **10a-c** or the *N*^1^,*N*^1^,*N*^8^,*N*^8^-tetrabenzylspermidine (**12**) in the presence of the coupling agent *O*-(benzotriazol-1-yl)-*N*,*N*,*N*′,*N*′-tetramethyluronium hexafluorophosphate (HBTU) and Et_3_N or ethyldiisopropylamine (DIEA) in dimethylformamide (DMF). The required diamine derivatives **10a-c** were obtained in the form of their bistrifluoroacetate salts from the corresponding linear, commercially available amino acids β-alanine (βAla, **8a**), γ-aminobutyric acid (γΑba, **8b**) and ε-aminocaproic acid (εAca, **8c**) through a four-step sequence, also depicted in Scheme 1. This involved the protection of the amino function with the triphenylmethyl (trityl, Trt) group [18,19] followed by coupling with dibenzylamine in the presence of HBTU and Et_3_N to give the amides **9a-c**. These were reduced with lithium aluminium hydride (LAH) in tetrahydrofuran (THF), and then the trityl group was removed by trifluoroacetic acid (TFA)-mediated acidolysis to provide compounds **10a-c**. On the other hand, the spermidine derivative **12** was synthesized from amide **9a** as follows. Compound **9a** was routinely detritylated with TFA and the thus-liberated primary amino function was acylated with succinic anhydride, in the presence of DIEA. Without isolation, the obtained intermediate was further coupled in a one-pot fashion with dibenzylamine in the presence of HBTU and DIEA and the thus-obtained trisamide **11** was finally reduced with LAH to give the tetrabenzylated SPD **12**. Experimental details for the syntheses described above and the complete characterization of intermediates and final products are provided in the Supplementary Material (Section S1).

### 2.2. Biological Evaluation

#### 2.2.1. Bacterial Strains and Cell Lines

The in vivo antibacterial activity of the conjugates was determined using the following three bacterial strains: wild-type *Escherichia coli* K12 (*E. coli* K12), *Escherichia coli* ΔTolC mutant strain (*E. coli* ΔTolC) lacking the TolC protein, which is involved in the efflux pumps operation, and wild-type *Staphylococcus aureus* (*S. aureus*). The anti-tumor activity of the conjugates was assessed using the human ZL34 and MeT5A cell lines, which were kindly offered by Professor G. Stathopoulos (University of Patras). ZL34 are cancer cells, derived from malignant pleural mesothelioma, while MeT5A are immortalized cells, which originate from healthy mesothelial tissue [20].

#### 2.2.2. In Vivo Antibacterial Activity

For the experimental procedures, the bacteria were cultured in luria broth (LB) medium (tryptone 10 g/L, yeast extract 5 g/L, NaCl 10 g/L) as previously described [10]. The bacteria cultures were grown in the presence of CAM or each conjugate at increasing concentration (1–100 μM). The cultures were incubated at 37 °C until the absorbance (OD_560_) of the reference culture in the absence of antibiotic reached the value of 0.7. At this point, the growth of the cultures was terminated by placing them in an ice bath, and the measurement of the optical density followed [10]. These measurements were used to calculate the half-maximal effective concentration (EC_50_) values [21].

#### 2.2.3. Affinity Measurement of PA–CAM Conjugates for the *E. coli* 70S Ribosome

Reassociated 70S ribosomes were prepared from *E. coli* K12 cells as described previously [22]. 70S ribosomes were incubated in buffer A (100 mM Tris-HCl pH 7.2, 100 mM ammonium acetate, 10 mM magnesium acetate, 6 mM β-mercaptoethanol) with 10 μΜ [^14^C]-chloramphenicol (150 dpm/pmol) at a final concentration of 0.20 μM [23]. After incubation for 10 min at 37 °C, the mixture was diluted with 3 mL of cold buffer A and filtered through a 25-mm diameter cellulose nitrate membrane filter (Millipore 0.45 μm pore size). The filter was washed three times with 3 mL of cold buffer A and the radioactivity which remained bound on the filter was measured. The binding of [^14^C]-chloramphenicol was studied in competition with CAM or PA–CAM conjugates by keeping the concentration of [^14^C]-CAM constant (10 μM) and increasing the concentration of non-radioactive conjugates [23].

#### 2.2.4. Evaluation of the Anticancer Activity 

The antitumor activity of the conjugates was assessed using the human ZL34 and MeT5A cell lines as previously described [10]. ZL34 and Met5A cells were plated in sterile 6-well microtiter plates and grown in Dulbecco’s modified Eagle’s medium (DMEM), supplemented with 10% heat-inactivated fetal bovine serum and 1% penicillin/streptomycin. Cultures were maintained in a humidified atmosphere with 5% CO_2_ at 37 °C. Compounds **3** and **4** were added at final concentrations of 30 and 60 μΜ, and then cells were grown for 24, 72 and 96 h. In parallel, solutions of conjugates combined with a ten-fold concentration of SPD were incubated under the same conditions. After treatment, the drug was removed by washing the cells twice with phosphate-buffered saline (PBS). The cells were then trypsinized (0.5 mL trypsin-EDTA×1 solution/well, 5 min at 37 °C), mixed with 1 mL DMEM and collected by centrifugation at 3000 × *g* for 5 min. Cell viabilities were determined by the trypan blue exclusion assay, using a TC10 automated cell counter (BIORAD) [10]. Viable cells were expressed as a percentage of total cells.

#### 2.2.5. Immunoblotting

Cell lysates were prepared after treatment with 60 μΜ of conjugates for 48 h. Total cellular proteins were isolated at 4 °C using RIPA buffer (ABCAM) and mixed with 4% SDS-containing loading buffer. Protein concentrations were determined using Bradford assay [24]. Lysates (100 mg protein/well) were reduced in Laemmli buffer containing 1 mM dithiothreitol, resolved by 10% SDS-PAGE and electrotransfered to Immobilon-P PVDF membranes (Millipore, Massachusetts, USA) according to standard procedures [25]. Blots were incubated at 4 °C overnight with rabbit monoclonal antibody against mitochondrial cytochrome c oxidase subunit II (COX2) (anti-mtCO2 antibody, ABCAM) at a 1:1000 dilution in blocking buffer. After washing with TBS-Tween and incubation with secondary antibody coupled to horse radish peroxidase, protein bands were visualized with ECL^TM^ chemiluminescence reagent (Amersham) and detected by autoradiography. The intensity of bands was quantified by Image Analysis, using the Image-Pro Plus 7 software (Media Cybernetics). Blots were re-probed with a mouse anti-actin antibody (Santa Cruz Biotechnology) to verify equal protein loading. 

#### 2.2.6. Quantification of the Intracellular Levels of Conjugates **3** and **4** Plus Polyamines

PAs and conjugates **3** and **4** in ZL34 and Met5A cells were determined by reverse phase high-performance liquid chromatography (RP-HPLC-DAD), as described previously [11]. Briefly, 100 mg cells (wet weight), either ZL34 or Met5A, which were grown in the presence of 60 μΜ of conjugates, were washed and then homogenized in 0.6 M HClO_4_. Cultures grown in the absence of the conjugates were used as reference. In the isolates, hexamethylenediamine dihydrochloride was added as an internal standard, followed by dansylation and subsequent extraction by toluene. The extracts were dried, reconstituted in acetonitrile/10 mM phosphate buffer pH 4.4 (45:55 *v*/*v*) and filtered through a 0.2-μm membrane (Spartan 13/0.2 RC Whatman GmbH, Dassel, Germany) prior to HPLC analysis. The HPLC system included a Varian Star 9010 solvent delivery system with a 250 × 4.6 mm Luna 5 μm C18(2) 100 A column (Phenomenex Co., USA) and a photodiode array detector (Shimadzu SPDM20A).

The gradient elution profile is presented in Table 1. The flow rate was set at 1.0 mL/min. Polyamines were quantified at 258 nm and the conjugates at 275 nm. The detection limits for PUT, SPD, and SPM were 52 pmol, 18 pmol, and 36 pmol at 258 nm, respectively. The detection limits for compounds **3** and **4** were 25 pmol and 30 pmol at 275 nm, respectively. All experiments were run in triplicate. The coefficients of variation (C.V.) of the intra-assays were 5–7%, while those of the inter-assays ranged from 13 to 17%. LC Solution Software (Shimadzu) was used for data processing and the results were expressed as nmol/mg protein.

#### 2.2.7. System Modeling and Molecular Dynamics Simulations

The binding modes of conjugates **3** and **4** in a region near the catalytic center (A2938, C2939) of the human mitochondrial large ribosomal subunit (hmLRS) have been modeled using molecular dynamics simulations. Three-dimensional models of the compounds were produced with Arguslab 4.0.1 (Planaria Software LLC, Seattle, WA, USA, http://www.arguslab.com), starting with the 3D structure of CAM derived from the 50S ribosomal subunit structure of *E. coli* in complex with CAM (PDB:3OFC). CHARMM force field parameters and topology files were generated by the SwissParam Tool [26]. The 3D structure of the hmLRS (PDB:3J7Y) with a resolution of 3.4 Å was used to model a ~28 Å radius environment around each analog near the catalytic center. The analogs were initially positioned with their CAM moieties within the drug crystallographic pocket mapped into the hmLRS according to the intersystem correspondence of large subunit relevant nucleotides shown in Table S1 of Supplementary Material S3. The systems were then solvated with ~10,000 TIP3 water molecules, and then neutralized with Mg^2+^ ions using the VMD program [27]. All systems derived as above were energy minimized and then subjected to short NPT molecular dynamics (MD) simulations to stabilize their volume at T = 300 K and P = 1 Atm. Subsequently, NVT MD simulations for 30 ns at 300 K, with the particle mesh Ewald (PME) algorithm, and the rigid bonds assignments were run using the NAMD software [28] and the CHARMM27 force field for proteins and nucleic acids. During MD simulations, harmonic constraints have been imposed to all nucleic acid backbone atoms. Finally, an average structure over the last 1 ns of each simulation trajectory was energy minimized and used for further analysis. All molecular visualizations were produced with the open-ource Pymol version 1.6 Copyright symb 2009-2013 Schrödinger, LLC.

#### 2.2.8. Statistics

All data presented in the figures and tables represent the mean value and standard deviation, obtained from at least two independently performed experiments. Statistical tests (data variability, t-tests, one-way ANOVA) were performed using the program IBM Statistics 24. The statistical significance threshold was set at *p* = 0.05.

## 3. Results

### 3.1. Antibacterial Activity of Novel PA–CAM Conjugates

All cells were cultivated in the presence of CAM or PA–CAM conjugates in concentrations reaching up to 100 μM. An *E. coli* strain was used as a model of Gram-negative bacteria, and an *S. aureus* strain was used as a model of Gram-positive bacteria. The antibacterial activity of the conjugates is expressed by the EC_50_ value (Table 2). With the exception of compound **3**, none of the new compounds exhibited inhibitory activity against the growth of bacterial cultures. Thus, either new derivatives failed to cross the bacterial cell membrane or they are non-active within the bacterial cells. The latter could be attributed either to new derivatives not binding on the bacterial ribosome, or binding without effect [29]. An alternative explanation would be that the conjugates may enter the bacteria but are exported by efflux pumps, the specific bacterial transporters which represent a resistance mechanism. However, since *E. coli* ΔTolC growth was not inhibited, such a possibility does not seem feasible. 

### 3.2. The New PA–CAM Conjugates Compete with CAM to Bind on the Bacterial Ribosome

To clarify whether the new PA–CAM conjugates maintain their binding site on the 70S bacterial ribosome, we carried out competition experiments. These data (Figure S1, Supplementary Material) allowed us to measure the concentration required for the 50% displacement of the radioactive CAM (IC_50_), in the presence of increasing concentrations of all PA–CAM conjugates. The affinity constants, *K_i_*, were calculated using the following Cheng–Prusoff relationship (1) [30] and their values are presented in Table 3. According to the values of Table 3, the decrease of the affinity for 70S ribosome follows the order **3**>**7**>**4**>**6**>**CAM**>**5**.

(1) $$K_{i}=\frac{{\mathrm{IC}}_{50}}{1+\frac{\left[I\right]}{K_{D}}}$$

where [*I*] is the concentration of [^14^C]-CAM (constant at 10 μM) and *K*_D_ is the dissociation constant of ribosome–chloramphenicol complex formation.

### 3.3. Antiproliferative Activity of Compound **4**

**Figure 2:**
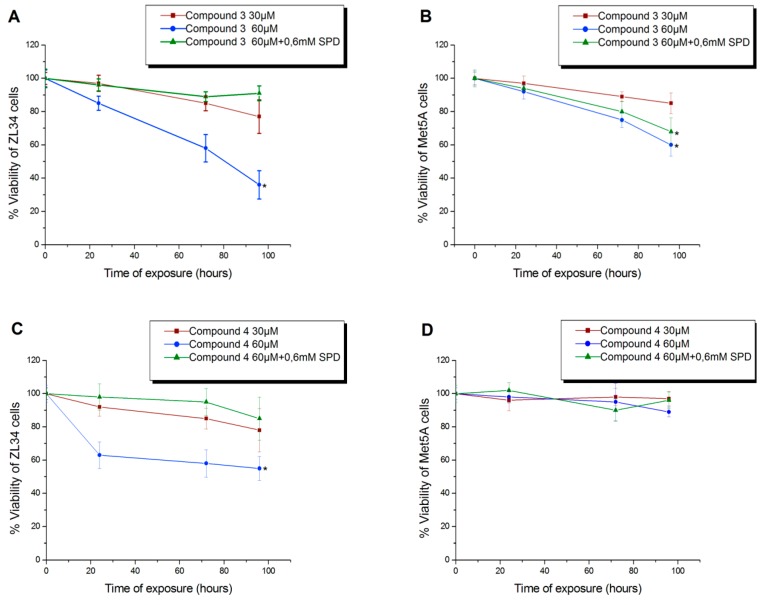
Cytotoxicity of compounds **3** (**A**,**B**) and **4** (**C**,**D**) on ZL34 mesothelioma cells and Met5A mesothelial immortalized cells. * Significantly different values from those measured in control samples (*p* < 0.05).

Based on the established antitumor activity of compound **3**, we were expecting that the addition of the benzyl groups on the spermidine domain of the compound could also affect this feature. Compound **4** demonstrated cytotoxicity for ZL34 cancer cells, which appeared to be inhibited when the cells were cultured in the presence of exogenous SPD in the medium (Figure 2C). Furthermore, conjugate **4** exhibited milder action against immortalized mesenchymal Met5A cells relative to conjugate **3**, as presented in Figure 2D. The other compounds **5**, **6** and **7** did not show any important antiproliferative activity.

### 3.4. Conjugates **3** and **4** Are Inhibitors of Mitochondrial Protein Synthesis

**Figure 3:**
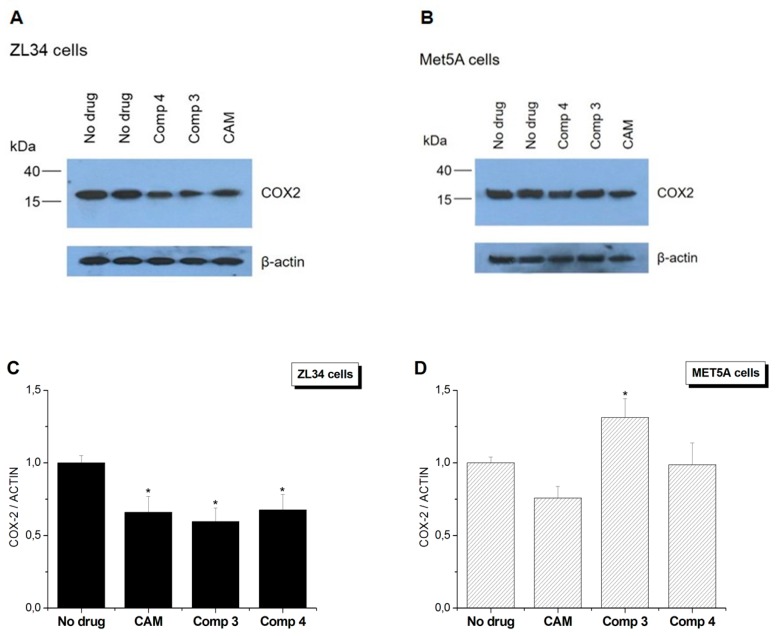
Effect of CAM or PA–CAM conjugates on cytoplasmic and mitochondrial protein synthesis. Western blot analysis on total protein extracts, derived from (**Α**) ZL34 and (**B**) Met5A cells. (**C**,**D**) Quantification of COX2 protein levels compared to actin levels after normalization. * Significantly different values from those measured in control samples (*p* < 0.05).

The cytotoxic effect of conjugates **3** and **4** on human cell lines triggered the investigation of the underlying mechanism. Taking into account the mitochondrial toxicity of CAM [31], we examined the possible disruption of mitochondrial protein synthesis. For this purpose, a mitochondrially encoded protein compartment of OXPHOS Complex IV, COX2 was measured. Indeed, as presented in Figure 3A, a remarkable decrease in COX2 levels was observed in ZL34 malignant cells. Simultaneously, actin levels, which were used as a reference of cytoplasmic protein synthesis level, did not demonstrate any decrease. In Figure 3C, data were normalized as to actin levels. COX2 levels were affected to a lesser extent in Met5A cells compared to ZL34 (Figure 3B,D).

### 3.5. Derivatives’ and Polyamines’ Intracellular Concentrations

**Figure 4:**
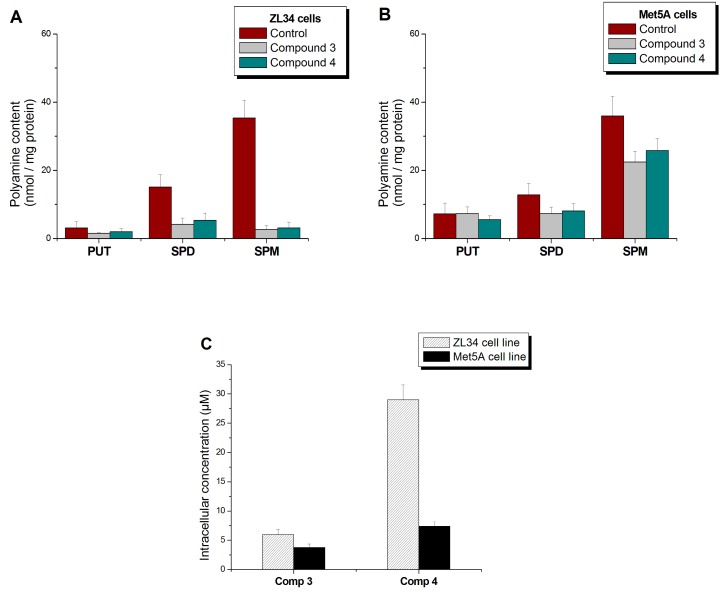
Changes in the polyamine levels of cells (**A**) ZL34 and (**B**) Met5A, after treatment with 60 μΜ of compound **3** or **4**. (**C**) Intracellular concentration of compounds **3** and **4**, detected in cell extracts.

The results of the identification and quantification of compounds **3** and **4** (for compound **4**, see also Figure S2, Supplementary Material) and PAs (Figure S3, Supplementary Material) are presented in Table 4. Regarding the ZL34 cancer cell line (Figure 4A), it appears that the presence of conjugates **3** and **4** resulted in the decrease of PUT, SPD and SPM levels, which was particularly pronounced in the case of SPM. In contrast, in immortalized Met5A cells (Figure 4B), in the presence of conjugates **3** and **4,** there was no remarkable change in PUT and SPD levels. SPM, however, showed a downward trend in immortalized cells, but to a lesser extent related to malignant cells. It is worth noting that conjugate **4** exhibited a remarkably high level of internalization in ZL34 cells relative to compound **3** (Figure 4C). Still, both conjugates were identified in higher concentrations in tumor ZL34 cells relative to Met5A mesothelial cells. The elevated polyamine intake rates of cancer cells could probably explain this observation [17].

### 3.6. System Modeling and Molecular Dynamics Simulations

**Figure 5:**
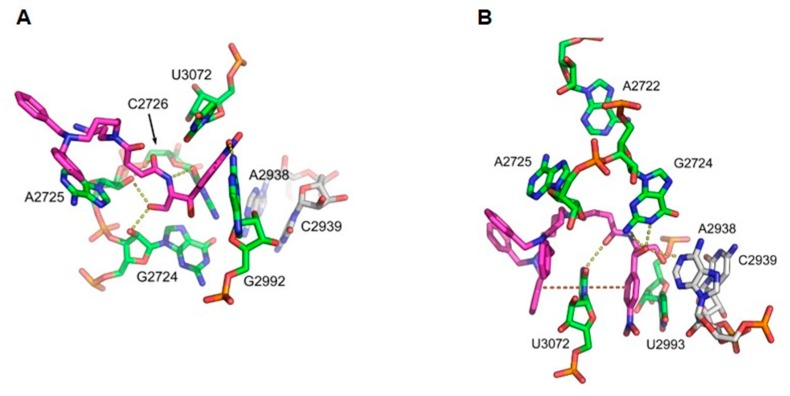
Molecular modeling of compounds binding mode in the vicinity of the catalytic center of hmLRS, derived from molecular dynamics (MD) simulations. Possible hydrogen bonds are shown with yellow bullets. For clarity only, relevant nucleotides of the environment are shown. Nucleotides A2938 and C2939 of the catalytic center are shown for orientation purposes in gray. (**A**) Compound **3**. Four possible hydrogen bonds stabilize compound **3** in the region, as shown in Table 5. (**B**) Compound **4**. Four possible hydrogen bonds (Table 5) as well as two stacking interactions stabilize compound **4** in the region. Note the hydrogen bond between N1 nitrogen of adenine in A2938 with a hydroxyl oxygen of compound **4** nearest to the CAM ring.

Based on the previous data, conjugates **3** and **4** seem to interact with mitochondrial protein synthesis, possibly by binding on the mitochondrial ribosome. Thus, we attempted to verify the possible stereochemical interactions of our compounds with the mitochondrial ribosome by applying molecular dynamics simulations. We also exploited our results from footprinting analysis on the 70S bacterial ribosome, taking into account the similarity of bacterial and mitochondrial ribosomal particles, as has been formulated by the theory of endosymbiosis [32]. Conjugates **3** and **4** demonstrated a similar footprinting pattern to CAM. Figure 5 illustrates the outcome of the simulations. Both compounds appear to form hydrogen bonds with G2724 of mitochondrial 16S rRNA, which corresponds to the G2061 of *E. coli* 23S rRNA. In addition, conjugate **3** interacts with A2725 and C2726 (A2062 and C2063, respectively), bases that lie within the CAM binding site on the bacterial ribosome. Furthermore, a hydrogen bond between conjugate **4** and U3072 (U2585 on 23S *E. coli* rRNA) suggests a contribution of the polyamine segment to the compound ribosomal binding affinity. Finally, the interactions of the conjugates with G2992 (conjugate **3**) and A2938 (conjugate **4**) indicate that they may enter the cleft of the peptidyl transferase center of the mitochondrial ribosome. 

## 4. Discussion

Bacterial resistance to antibiotics poses a serious risk to public health. Resistance can be developed by a variety of mechanisms, such as modifications on the antibiotic’s binding site [35], reduced membrane permeability [36,37], modifications on the antibiotic moiety [38] or increased efflux through specific transporters (efflux pumps) [39,40]. Despite ongoing research efforts, only a small number of new antibacterial compounds have been introduced into clinical practice over the last years, rendering resistance a serious therapeutic problem. An approach to overcome this issue is the chemical modification of already known effective antibiotics [41] and led us to focus on CAM derivatization [10,11,21,42,43,44].

Our new PA–CAM conjugates were tested either in Gram-positive or in Gram-negative bacteria and additionally in the mutant *E. coli* strain ΔTolC, which is more sensitive to many antibiotics. According to our results, neither compound inhibited bacterial growth, although both can be bound on *E. coli* ribosomes with binding affinities similar to CAM (Table 3). From the data of Table 3, it is obvious that all compounds can bind to the *E.coli* ribosomes with almost identical affinities. Neither the addition of two more benzyl groups on the lead molecule, nor the deletion of the aminopropyl moiety nor PA carbon skeleton modification changed the order of magnitude of their binding affinity, presented by the *K_i_* value. Moreover, while their binding affinities for the ribosome are high enough, none of them exerted important antimicrobial activity. Taking also into account the fact that the ΔTolC strain was not inhibited in the presence of our conjugates, it remains to be further clarified whether the bound conjugates inhibit ribosome function in vitro. Such a result will allow us to clarify their ability or inability to pass through the cell membrane. 

Multiple reports have been published concerning the undesirable side effects following administration of CAM, which are due to antibiotic activity on the mitochondrial ribosome [4]. This finding has prompted the investigation of the potential anticancer action of the antibiotic, as tumor cells are particularly dependent on mitochondrial activity due to their increased energy needs [3]. Along with increased energy requirements, the need for active metabolites is also elevated in tumor cells [45]. Among these metabolites are PAs, essential molecules for cell growth and survival. Indeed, cancer cells have to cope with this need by increasing both the biosynthesis and import of PAs into the cell [46]. The increased activity of PATs in cancer cells is a distinctive element, compared to normal cells, that may be useful in the specialized targeting of neoplastic cells [17]. 

These observations prompted us to investigate the antitumor activity of the PA–CAM conjugates. Interestingly, previous studies have established the antitumor potential of compound **3** [10]. Among the new compounds tested, only compound **4** exhibited a similar antiproliferative behavior to compound **3**, decreasing the viability of cancer cells and having a milder effect on Met5A non-tumor cells. 

Interestingly, compound **4** demonstrated elevated internalization in cancer cells compared to **3**, possibly due to an increased lipophilicity, derived from the two additional benzyl groups. Nevertheless, both compounds were identified in higher concentrations in ZL34 mesothelioma cells, exploiting the presence of more active transporters on cancer cell membranes [17]. Both derivatives are considered to enter the human cells using the PATs, since they competed with SPD, when the latter was added exogenously in the culture medium (Figure 2A,C).

CAM has been shown to act on mitochondria, inhibiting mitochondrial protein synthesis [47]. A hypothesis that we examined is the possibility that compounds **3** and **4** affect the viability of human cells in the same way. For this purpose, COX2 protein was used as a marker. COX2 is encoded by mitochondrial DNA and is translated with mitochondrial ribosomes [48]. Indeed, conjugates **3** and **4** caused a significant decrease in COX2 expression in tumor cells, while actin level, which was used as an indicator of cytoplasmic protein synthesis, remained unaffected, hinting that the mitoribosome may be the main target in the human cancer cells. Molecular dynamics simulations (Figure 5), indicate the placement of compounds on the mitochondrial ribosome in a way that could possibly inhibit the protein synthesis. Both compounds appear to form hydrogen bonds with nucleotides of mitochondrial 16S rRNA of the large ribosome subunit, which could justify the inhibition of mitochondrial protein synthesis.

PA levels in the tumor cells cultured in the presence of the two conjugates showed a remarkable decrease. A possible explanation for this is that the presence of PA–CAM conjugates, combined with the already elevated levels of cell PAs, triggers the activity of SSAT (spermidine/spermine N^1^-acetyltransferase), which is responsible for the first step of PA catabolism. SSAT, being unable to act on the conjugates, would, however, deplete only the endogenous PAs, thereby disrupting PA homeostasis [49]. This hypothesis is strengthened by the observation that PUT levels were not affected, since PUT is not used by SSAT as a substrate [50]. Another suggestion is that PA–CAM conjugates functioned in a competitive manner, lowering the intracellular levels of the PAs. 

## 5. Conclusions

In conclusion, the novel PA–CAM conjugates, synthesized with the aim of improving the activity of a previously developed SPD–CAM conjugate (**3**), did not demonstrate improved antibacterial activity, possibly due to their inability to penetrate the bacterial cell membrane. However, one of them, namely compound **4,** carrying two additional benzyl groups, proved to be effective against human mesothelioma ZL34 cells, while having a milder effect on Met5A cells. Therefore, this new compound could be considered as a promising antitumor agent. Moreover, the absence of antibacterial activity could render the compound as a therapeutic agent with no unintentional disturbance of the bacterial microflora of the patient, a necessary feature for the maintenance of homeostasis and normal function, which is often a side effect of orally-administrated drugs [51]. Finally, the deletion of the aminopropyl moiety of the lead molecule rendered it inactive either as an antibacterial or an antitumor agent, while the additional modification of the polyamine carbon skeleton did not further modify their limited biological activity.

## Figures and Tables

**Table 1:** Gradient elution profile. Mobile phase A: acetonitrile; mobile phase B: 10 mM phosphate buffer (pH 4.4).

Time (min)	Mobile Phase A (%)	Mobile Phase B (%)
0	45	55
14	80	20
15	90	10

**Table 2:** In vivo antibacterial activity of CAM and PA–CAM conjugates against wild-type (WT) and mutant strains of bacteria, expressed by the half-maximal effective concentration (EC_50_) value. Data represent the mean ± SD values obtained from three independent experiments.

Compound	EC_50_ (μΜ)		
	*Escherichia coli* K12	*Escherichia coli* ΔTolC	*Staphylococcus aureus*
**CAM**	6.2 ± 0.5	2.3 ± 0.6	3.4 ± 0.3
**3**	11.0 ± 0.9	8.9 ± 0.7	6.8 ± 0.5
**4**	>200	>200	>200
**5**	>200	>200	>200
**6**	>200	>200	>200
**7**	>200	>200	>200

**Table 3:** The binding affinity constants *K*_i_ of all PA–CAM.

Compound	*K_i_* (μΜ)
**CAM**	1.5 ± 0.1
**3**	0.8 ± 0.1
**4**	1.1 ± 0.1
**5**	2.6 ± 0.3
**6**	1.3 ± 0.2
**7**	1.0 ± 0.1

**Table 4:** Intracellular compound and polyamine concentrations (expressed nmol/mg protein). Cell lines ZL34 and Met5A were treated with 60 μΜ of compounds **3** and **4**. Values presented as mean ± SD, obtained from three independent repetitions.

			(nmol/mg Protein)		
Cell line	Compound	Concentration (μΜ)	*PUT*	*SPD*	*SPM*
**ZL34**	**-**	-	3.18 ± 1.82	15.16 ± 3.63	35.40 ± 5.16
	**3**	5.96 ± 0.92	1.58 ± 0.13	4.18 ± 1.87 ^1^	2.71 ± 1.17 ^2^
	**4**	29.00 ± 2.53	2.07 ± 0.91	5.36 ± 2.15 ^1^	3.17 ± 1.70 ^2^
**Met5A**	**-**	-	7.29 ± 3.17	12.87 ± 3.34	35.96 ± 11.72
	**3**	3.75 ± 0.60	7.38 ± 1.93	7.38 ± 1.85	22.50 ± 3.11
	**4**	7.40 ± 0.80	5.60 ± 1.10	8.14 ± 2.16	25.80 ± 6.57

**^1^** Significantly different values from those measured in control samples (*p* < 0.05); **^2^** Significantly different values from those measured in control samples (*p* < 0.01).

**Table 5:** Hydrogen bond pairs^$^ between atoms of mitochondrial rRNA and of compounds **3** and **4**^#^. Corresponding bases of *E. coli* 23S rRNA are noted in parenthesis. The matching of RNA nucleotides was based on data from Brown et al. [33].

rRNA	Comp. 3	rRNA	Comp. 4
N2: G2992 (G2505)	O1	N3: U3072 (U2585)	O5
O2: C2726 (C2063)	N2	N2: G2724 (G2061)	O3
O2′: G2724 (G2061)	O4	N1: G2724 (G2061)	O3
O2′: A2725 (A2062)	O4	N1: A2938 (A2451)	O3

**^$^** Hydrogen bonds have been assigned using the program Swiss-PdbViewer 4.0.1 [34]. **^#^** Atom numbering in the models of compounds **3** and **4** is given by Arguslab and is shown in Figure S4, Supplementary Material.

## Supplementary Materials

The following are available online at http://www.mdpi.com/2079-6382/8/1/9/s1, Experimental Section (S1): Experimental details for the synthesis and characterization of intermediate and final products; Results Section (S2): Figure S1: Competitive binding of [^14^C]-CAM with unlabeled CAM and compounds **3**–**7**, Figure S2: RP-HPLC chromatograms for the intracellular concentration of compound **4** in ZL34 and Met5A cells, Figure S3: RP-HPLC chromatogram of dansylated PAs in ZL34 cells, Figure S4: Atom numbering in compound **3** and **4** models; Materials and Methods Section (S3): Table S1: Intersystem Correspondence of Relevant Large Subunit Nucleotides.

## References

[1] Dinos G., Athanassopoulos C., Missiri D., Giannopoulou P., Vlachogiannis I., Papadopoulos G., Papaioannou D., Kalpaxis D. (2016). Chloramphenicol Derivatives as Antibacterial and Anticancer Agents: Historic Problems and Current Solutions. Antibiotics.

[2] Yuan Z.R., Shi Y. (2008). Chloramphenicol induces abnormal differentiation and inhibits apoptosis in activated T cells. Cancer Res..

[3] Lamb R., Ozsvari B., Lisanti C.L., Tanowitz H.B., Howell A., Martinez-Outschoorn U.E., Sotgia F., Lisanti M.P. (2015). Antibiotics that target mitochondria effectively eradicate cancer stem cells, across multiple tumor types: Treating cancer like an infectious disease. Oncotarget.

[4] Singh R., Sripada L., Singh R. (2014). Side effects of antibiotics during bacterial infection: Mitochondria, the main target in host cell. Mitochondrion.

[5] Marks J., Kannan K., Roncase E.J., Klepacki D., Kefi A., Orelle C., Vázquez-Laslop N., Mankin A.S. (2016). Context-specific inhibition of translation by ribosomal antibiotics targeting the peptidyl transferase center. Proc. Natl. Acad. Sci. USA.

[6] Schlünzen F., Zarivach R., Harms J., Bashan A., Tocilj A., Albrecht R., Yonath A., Franceschi F. (2001). Structural basis for the interaction of antibiotics with the peptidyl transferase centre in eubacteria. Nature.

[7] Dunkle J.A., Xiong L., Mankin A.S., Cate J.H.D. (2010). Structures of the Escherichia coli ribosome with antibiotics bound near the peptidyl transferase center explain spectra of drug action. Proc. Natl. Acad. Sci. USA.

[8] Bulkley D., Innis C.A., Blaha G., Steitz T.A. (2010). Revisiting the structures of several antibiotics bound to the bacterial ribosome. Proc. Natl. Acad. Sci. USA.

[9] Vázquez-Laslop N., Mankin A.S. (2018). Context-Specific Action of Ribosomal Antibiotics. Annu. Rev. Microbiol..

[10] Kostopoulou O.N., Kouvela E.C., Magoulas G.E., Garnelis T., Panagoulias I., Rodi M., Papadopoulos G., Mouzaki A., Dinos G.P., Papaioannou D. (2014). Conjugation with polyamines enhances the antibacterial and anticancer activity of chloramphenicol. Nucleic Acids Res..

[11] Magoulas G.E., Kostopoulou O.N., Garnelis T., Athanassopoulos C.M., Kournoutou G.G., Leotsinidis M., Dinos G.P., Papaioannou D., Kalpaxis D.L. (2015). Synthesis and antimicrobial activity of chloramphenicol-polyamine conjugates. Bioorganic Med. Chem..

[12] Dever T.E., Ivanov I.P. (2018). Roles of polyamines in translation. J. Biol. Chem..

[13] Igarashi K., Kashiwagi K. (2018). Effects of polyamines on protein synthesis and growth of Escherichia coli. J. Biol. Chem..

[14] Shah P., Swiatlo E. (2008). A multifaceted role for polyamines in bacterial pathogens. Mol. Microbiol..

[15] Wallace H.M., Fraser A.V., Hughes A. (2003). A perspective of polyamine metabolism. Biochem. J..

[16] Paz E.A., Lafleur B., Gerner E.W. (2014). Polyamines are oncometabolites that regulate the LIN28/let-7 pathway in colorectal cancer cells. Mol. Carcinog..

[17] Palmer A.J., Wallace H.M. (2010). The polyamine transport system as a target for anticancer drug development. Amino Acids.

[18] Tsiakopoulos N., Damianakos C., Karigiannis G., Vahliotis D., Papaioannou D., Sindona G. (2002). Syntheses of crowned polyamines using isolable succinimidyl esters of N-tritylated linear amino acids and peptides. Arkivoc.

[19] Magoulas G.E., Garnelis T., Athanassopoulos C.M., Papaioannou D., Mattheolabakis G., Avgoustakis K., Hadjipavlou-Litina D. (2012). Synthesis and antioxidative/anti-inflammatory activity of novel fullerene-polyamine conjugates. Tetrahedron.

[20] Ke Y., Reddel R.R., Gerwin B.I., Reddel H.K., Somers A.N., McMenamin M.G., LaVeck M.A., Stahel R.A., Lechner J.F., Harris C.C. (1989). Establishment of a human in vitro mesothelial cell model system for investigating mechanisms of asbestos-induced mesothelioma. Am. J. Pathol..

[21] Kostopoulou O.N., Magoulas G.E., Papadopoulos G.E., Mouzaki A., Dinos G.P., Papaioannou D., Kalpaxis D.L. (2015). Synthesis and evaluation of chloramphenicol homodimers: Molecular target, antimicrobial activity, and toxicity against human cells. PLoS ONE.

[22] Xaplanteri M.A., Petropoulos A.D., Dinos G.P., Kalpaxis D.L. (2005). Localization of spermine binding sites in 23S rRNA by photoaffinity labeling: Parsing the spermine contribution to ribosomal 50S subunit functions. Nucleic Acids Res..

[23] Bougas A., Vlachogiannis I.A., Gatos D., Arenz S., Dinos G.P. (2017). Dual effect of chloramphenicol peptides on ribosome inhibition. Amino Acids.

[24] Bradford M.M. (1976). A rapid and sensitive method for the quantitation of microgram quantities of protein utilizing the principle of protein-dye binding. Anal. Biochem..

[25] Wang B., Ao J., Yu D., Rao T., Ruan Y., Yao X. (2017). Inhibition of mitochondrial translation effectively sensitizes renal cell carcinoma to chemotherapy. Biochem. Biophys. Res. Commun..

[26] Zoete V., Cuendet M.A., Grosdidier A., Michielin O. (2011). SwissParam: A fast force field generation tool for small organic molecules. J. Comput. Chem..

[27] Humphrey W., Dalke A., Schulten K. (1996). VMD: Visual molecular dynamics. J. Mol. Graph..

[28] Phillips J.C., Braun R., Wang W., Gumbart J., Tajkhorshid E., Villa E., Chipot C., Skeel R.D., Kalé L., Schulten K. (2005). Scalable molecular dynamics with NAMD. J. Comput. Chem..

[29] Tereshchenkov A.G., Dobosz-Bartoszek M., Osterman I.A., Marks J., Sergeeva V.A., Kasatsky P., Komarova E.S., Stavrianidi A.N., Rodin I.A., Konevega A.L. (2018). Binding and Action of Amino Acid Analogs of Chloramphenicol upon the Bacterial Ribosome. J. Mol. Biol..

[30] Cheng Y.-C., Prusoff W.H. (1973). Relationship between the inhibition constant (*K_I_*) and the concentration of inhibitor which causes 50 per cent inhibition (*I_50_*) of an enzymatic reaction. Biochem. Pharmacol..

[31] Tian F., Wang C., Tang M., Li J., Cheng X., Zhang S., Ji D., Huang Y., Li H., Tian F. (2014). The antibiotic chloramphenicol may be an effective new agent for inhibiting the growth of multiple myeloma. Oncotarget.

[32] Andersson S.G.E., Zomorodipour A., Andersson J.O., Sicheritz-Pontén T., Alsmark U.C.M., Podowski R.M., Näslund A.K., Eriksson A.S., Winkler H.H., Kurland C.G. (1998). The genome sequence of Rickettsia prowazekii and the origin of mitochondria. Nature.

[33] Brown A., Amunts A., Bai X.C., Sugimoto Y., Edwards P.C., Murshudov G., Scheres S.H.W., Ramakrishnan V. (2014). Structure of the large ribosomal subunit from human mitochondria. Science.

[34] Guex N., Peitsch M.C. (1997). SWISS-MODEL and the Swiss-PdbViewer: An environment for comparative protein modeling. Electrophoresis.

[35] Douthwaite S. (1992). Functional interactions within 23S rRNA involving the peptidyltransferase center. J. Bacteriol..

[36] Delcour A.H. (2009). Outer membrane permeability and antibiotic resistance. Biochim. Biophys. Acta.

[37] Mortimer P.G.S., Piddok L.J.V. (1993). The accumulation of five antibacterial agents in porin-deficient mutants of escherichia coli. J. Antimicrob. Chemother..

[38] Murray I.A., Shaw W.V. (1997). O-Acetyltransferases for chloramphenicol and other natural products. Antimicrob. Agents Chemother..

[39] Okusu H., Ma D., Nikaido H. (1996). AcrAB efflux pump plays a major role in the antibiotic resistance phenotype of Escherichia coli multiple-antibiotic-resistance (Mar) mutants. J. Bacteriol..

[40] Ghisalberti D., Masi M., Pagès J.-M., Chevalier J. (2005). Chloramphenicol and expression of multidrug efflux pump in Enterobacter aerogenes. Biochem. Biophys. Res. Commun..

[41] Dinos G.P. (2017). The macrolide antibiotic renaissance. Br. J. Pharmacol..

[42] Drainas D., Kalpaxis D.L., Coutsogeorgopoulos C. (1987). Inhibition of ribosomal peptidyltransferase by chloramphenicol: Kinetic studies. Eur. J. Biochem..

[43] Drainas D., Mamos P., Coutsogeorgopoulos C. (1993). Aminoacyl Analogs of Chloramphenicol: Examination of the Kinetics of Inhibition of Peptide Bond Formation. J. Med. Chem..

[44] Michelinaki M., Mamos P., Coutsogeorgopoulos C., Kalpaxis D.L. (1997). Aminoacyl and peptidyl analogs of chloramphenicol as slow-binding inhibitors of ribosomal peptidyltransferase: A new approach for evaluating their potency. Mol Pharmacol.

[45] Hanahan D., Weinberg A.R. (2011). Hallmarks of Cancer: The Next Generation. Cell.

[46] Agostinelli E., Marques M.P.M., Calheiros R., Gil F.P.S.C., Tempera G., Viceconte N., Battaglia V., Grancara S., Toninello A. (2010). Polyamines: Fundamental characters in chemistry and biology. Amino Acids.

[47] Kim H.J., Maiti P., Barrientos A. (2017). Mitochondrial ribosomes in cancer. Semin. Cancer Biol..

[48] Yusoff A.A.M. (2015). Role of mitochondrial DNA mutations in brain tumors: A mini-review. J. Cancer Res. Ther..

[49] Wallace H.M., Niiranen K. (2007). Polyamine analogues—An update. Amino Acids.

[50] Pegg A.E. (2008). Spermidine/spermine-N(1)-acetyltransferase: A key metabolic regulator. Am. J. Physiol. Endocrinol. Metab..

[51] Lange K., Buerger M., Stallmach A., Bruns T. (2016). Effects of Antibiotics on Gut Microbiota. Dig. Dis..

