# Supplementary material for: New Chloramphenicol Derivatives from the Viewpoint of Anticancer and Antimicrobial Activity
Source: Antibiotics (Basel). 2019 Jan 29;8(1):9. doi: 10.3390/antibiotics8010009 (PMC6466596; doi:10.3390/antibiotics8010009)
Supplement: Supplementary file 1 [file antibiotics-08-00009-s001.pdf]

New Chloramphenicol Derivatives from the Viewpoint of Anticancer and Antimicrobial Activity

Panagiota C. Giannopoulou [1], Dionissia A. Missiri [2], Georgia G. Kournoutou[1], Eleni Sazakli[3], Georgios E. Papadopoulos [4], Dionissios Papaioannou [2], George P. Dinos [1,*], Constantinos M. Athanassopoulos [2,*] and Dimitrios L. Kalpaxis [1,†]

[1] Department of Biochemistry, School of Medicine, University of Patras, GR-26504 Patras, Greece; p.giannopoulou@upnet.gr (P.C.G.); gkurnutu@upatras.gr (G.G.K.); dim.kalpax@gmail.com (D.L.K.)
[2] Laboratory of Synthetic Organic Chemistry, Department of Chemistry, University of Patras, GR-26504 Patras, Greece; dionysia.misiri@gmail.com (D.A.M.); dapapaio@upatras.gr (D.P.)
[3] Laboratory of Public Health, School of Medicine, University of Patras, 26504 Patras, Greece; esazakli@med.upatras.gr
[4] Department of Biochemistry & Biotechnology, University of Thessaly, Biopolis, GR-41500 Larissa, Greece; geopap@uth.gr
† Dedicated in memory of the late Prof. Dimitrios L. Kalpaxis
* Correspondence: dinosg@upatras.gr (G.P.D.); kath@chemistry.upatras.gr (C.M.A.); Tel.: +30-261-096-9125 (G.P.D.), +30-261-099-7909 (C.M.A.)

Received: 19 December 2018; Accepted: 23 January 2019; Published: date

Supplementary Material

Contents

S1. Experimental section 1
S1.1 Chemistry 1
S1.1.1 General synthetic 1
S1.1.2 General procedure for the preparation of *N*-tritylated amino acids 2
S1.1.3 General procedure for the preparation of N-tritylated amides 9a-c 2
S1.1.4 General procedure for the LAH-mediated reduction of *N*-tritylated amides 9a –c. 3
Preparation of substituted diamines TrtNH-$(CH_2)_3$-NBn_2, TrtNH-$(CH_2)_4$-NBn_2 and TrtNH-$(CH_2)_6$-NBn_2 3
S1.1.5 General procedure for the preparation of the N^1,N^1-dibenzylated diamines as their corresponding bistrifluoroacetate salts 10 4
S1.1.6 Synthesis of N^1,N^1,N^8,N^8-tetrabenzyl-4-aza-5-oxaoctadiamide (11) 5
S1.1.7 Synthesis of N^1,N^1,N^8,N^8-tetrabenzylspermidine (12) 6
S1.1.8 General procedure for the synthesis of the PA-CAM conjugates 4-7 7
S2. Results section 9
S3. Materials and Methods Section 11

S1. Experimental section

S1.1. Chemistry

S1.1.1. General synthetic

Melting points were determined with a Buchi SMP-20 apparatus and are uncorrected. Attenuated total reflection (ATR)-IR spectra were recorded for KBr pellets or neat samples on a FT-IR Bruker EQUINOX55 spectrometer. ^{1}H-NMR spectra were obtained at 400.13 or 600.13 MHz and ^{13}C-NMR spectra at 100.62 or 150.9 MHz on Bruker Avance 400-DPX or AvanceIII HD spectrometers, respectively. Chemical shifts (δ) for $CDCl_3$ or CD_3OD solutions are reported in units, parts per million

(ppm) downfield from TMS. Electron-spray ionization (ESI) mass spectra were recorded at 30 eV, on a Micromass-Platform LC spectrometer using MeOH as solvent.

Flash column chromatography (FCC) was performed on Merck silica gel 60 (230-400 mesh) and TLC on 60 Merck $60F_{254}$ films (0.2 mm) precoated on aluminium foil. Spots were visualized with UV light at 254 nm and by spraying with a ninhydrine solution (0.3 g ninhydrin, 3 mL gl. acetic acid, 97 mL 2-propanol) and/or charring agent [10 g $(NH_4)_2SO_4$, 5 mL conc. H_2SO_4, 95 mL H_2O]. The solvent systems used for the development of TLC or FCC are the following: (A) PhMe/EtOAc (99:1), (B) PhMe/EtOAc (97:3), (C) PhMe/EtOAc (95:5), (D) PhMe/EtOAc (9:1), (E) PhMe/EtOAc (8:2), (F) PhMe/EtOAc (7:3), (G) PhMe/EtOAc (1:1), (H) $CHCl_3$/MeOH (95:5), (I) $CHCl_3$/MeOH (9:1), (J) $CHCl_3$/MeOH (1:1), (K) $CHCl_3$/MeOH/Et_3N (95:5:0.5), (L) $CHCl_3$/MeOH/Et_3N (8:2:1), (M) $CHCl_3$/MeOH/AcOH (85:10:5), (N) $CHCl_3$/MeOH/conc. NH_3 (9:2.5:0.2).

All solvents were dried and/or purified according to standard procedures prior to use. All reagents employed in the present work were purchased from either Aldrich or Fluka or TCI-Europe or Alfa Aesar or Acros and were used without further purification.

S1.1.2. General procedure for the preparation of *N*-tritylated amino acids

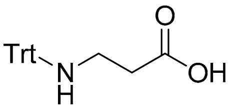

Trt-βAla

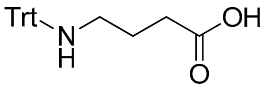

Trt-γAba

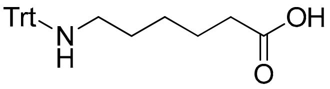

Trt-εAca

The preparation of *N*-trityl-β-alanine (Trt-βAla) and *N*-trityl-γ-aminobutyric acid (Trt-γAba) from the corresponding amino acids β-alanine (**8a**) and γ-aminobutyric acid (**8b**) has been described by our research group [18, 19]. The *N*-trityl-ε-aminocaproic acid (**8c**) was obtained by using the procedure reported for the preparation of Trt-βAla [18].

S1.1.2.1. *N*-Trityl-ε-aminocaproic acid (Trt-εAca)

Yellowish oil; Yield: 85%; R*f* (G): 0.38.
ATR-IR (cm^{-1}): 2934, 1702, 763, 744, 700
MS (ESI, 30 eV): *m/z* 396.59 [M+Na], 374.55 [M+H], 243.73 [Trt].
^{1}H-NMR (400.13 MHz, $CDCl_3$, δ): 7,49 (6H, d, J = 7.6 Hz), 7.30-7.25 (6H, m), 7.21-7.18 (3H, m), 2.37 (2H, br.s), 2.32 (2H, t, J = 7.2 Hz), 2.18 (2H, t, J = 6.8 Hz), 1.63-1.48 (4H, m), 1.39-1.31 (2H, m) ppm.
^{13}C-NMR (100.61 MHz, $CDCl_3$, δ): 179.8, 145.8 (3C), 128.7 (6C), 127.8 (6C), 126.3 (3C), 71.2, 43.6, 34.2, 30.2, 26.8, 24.7 ppm.

S1.1.3. General procedure for the preparation of N-tritylated amides 9a-c

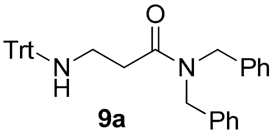

9a

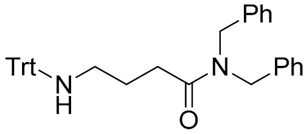

9b

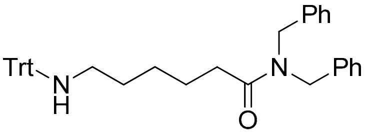

9c

To a solution of Trt-βAla or Trt-γAba or Trt-εAca (2.2 mmol) in anhydrous CH_2Cl_2 (3.3 mL), kept at 0 ^oC, was added sequentially $(PhCH_2)_2NH$ (0.47 mL, 2.42 mmol), Et_3N (0.92 mL, 6.62 mmol) and HBTU (0.92 g, 2.42 mmol). The reaction mixture was stirred at 0 ^oC for 20 min and at ambient temperature for the time indicated below and then diluted with CH_2Cl_2 and washed sequentially with 5% aqueous $NaHCO_3$, H_2O, ice-cold 5% aqueous citric acid, H_2O and brine. The organic phase was

then dried over anhydrous Na_2SO_4 and the solvent evaporated under reduced pressure. Amide **9b** was obtained pure upon triturating the residue with cold Et_2O and filtration of the precipitate under vacuo. Amide **9a** was obtained pure after FCC and using solvent system E for elution. Finally, amide **9c** was obtained pure also after FCC and using solvent system C initially, followed by solvent system D, for elution.

S1.1.3.1. *N*,*N*-Dibenzyl-3-tritylaminopropanamide (9a)

Reaction time: 6 h; White solid; Yield: 80%; M.p. 113.5-115.5 °C; R*f* (D): 0.44.
ATR-IR (cm^{-1}): 3315, 1630, 753, 697.
MS (ESI, 30 eV): *m*/*z* 1043.30 [2M+Na], 549.38 [M+K], 533.42 [M+Na], 511.51 [M+H], 243.44 [Trt].
^{1}H-NMR (400.13, $CDCl_3$, *δ*): 7.51-7.49 (6H, d, *J* = 6.8 Hz) and 7.41-7.20 (19H, three m), 4.63 (2H, s), 4.49 (2H, s), 2.66 (2H, unresolv. t), 2.51 (2H, unresolv. t), 1.60 (1H, br. s) ppm.
^{13}C-NMR (100.61, $CDCl_3$, *δ*): 172.7, 146.2 (3C), 137.2, 136.5, 129.0 (2C), 128.7 (6C), 128.6 (2C), 128.3 (2C), 127.8 (6C), 127.4, 127.2, 126.4 (3C), 126.2 (2C), 71.0, 49.9, 48.2, 39.9, 34.1 ppm.

S1.1.3.2. *N*,*N*-Dibenzyl-4-tritylaminobutanamide (9b)

Reaction time: 4 h; White solid; Yield: 65%; M.p. 133-135 °C; R*f* (C): 0.19.
ATR-IR (cm^{-1}): 3304, 1626, 750, 698.
MS (ESI, 30 eV): *m*/*z* 1071.41 [2M+Na], 563.30 [M+K], 547.34 [M+Na], 525.50 [M+H], 243.50 [Trt].
^{1}H-NMR (400.13, $CDCl_3$, *δ*): 7.46 (6H, d, *J* = 7.2 Hz), 7.38-7.16 (19H, m), 4.63 (2H, s), 4.45 (2H, s), 2.53 (2H, t, *J* = 7.6 Hz), 2.19 (2H, q, *J* = 8.0 Hz), 1.93 (2H, quint. *J* = 8 Hz), 1.63 (1H, t, *J* = 8.0 Hz) ppm.
^{13}C-NMR (100.61, $CDCl_3$, *δ*): 173.5, 146.2 (3C), 137.5, 136.6, 129.0 (2C), 128.6 (8C), 128.3 (2C), 127.8 (6C), 127.6, 127.4, 126.4 (3C,), 126.2 (2C), 70.8, 49.9, 48.2, 43.2, 31.3, 26.5 ppm.

S1.1.3.3. *N*,*N*-Dibenzyl-6-tritylaminohexanamide (9c)

Reaction time: 1.5 h; Pale yellow oil; Yield: 65%; M.p. 133-135 °C; Rf (C): 0.23.
ATR-IR (cm^{-1}): 3310, 1635, 750, 697.
MS (ESI, 30 eV): m/z 575.51 [M+Na], 243.64 [Trt].
^{1}H-NMR (400.13, $CDCl_3$, δ): 7.44 (6H, d, *J* = 7.6 Hz,), 7.34-7.10 (19H, three m), 4.56 (2H, s), 4.43 (2H, s), 2.35 (2H, q, *J* = 7.6 Hz), 2.08 (2H, unresolv. t), 1.65 (2H, quint., *J* = 7.6 Hz), 1.60 (1H, br. s), 1.50 (2H, quint., *J* = 7.6 Hz), 1.36 (2H, quint., *J* = 7.6 Hz) ppm.
^{13}C-NMR (100.61, $CDCl_3$, δ): 173.6, 146.2 (3C), 137.5, 136.6, 129.0 (2C), 128.7 (6C), 128.6 (2C), 128.3 (2C), 127.8 (6C), 127.6, 127.4, 126.4 (3C), 126.2 (2C), 70.9, 49.9, 48.1, 43.5, 33.2, 30.7, 27.2, 25.4 ppm.

S1.1.4. General procedure for the LAH-mediated reduction of *N*-tritylated amides 9a –c.

Preparation of substituted diamines TrtNH-$(CH_2)_3$-NBn_2, TrtNH-$(CH_2)_4$-NBn_2 and TrtNH-$(CH_2)_6$-NBn_2

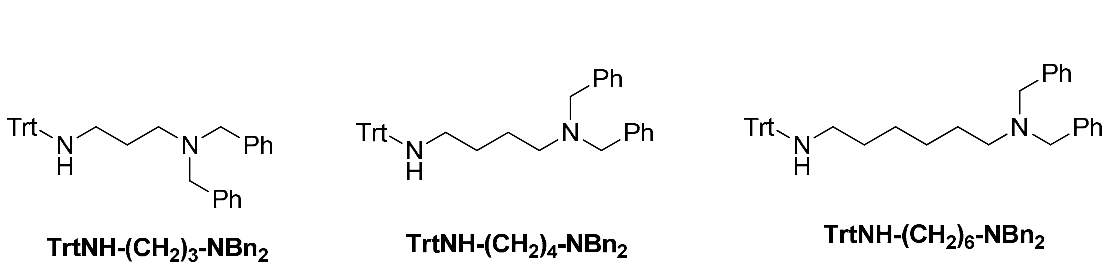

TrtNH-$(CH_2)_3$-NBn_2 **TrtNH-$(CH_2)_4$-NBn_2** **TrtNH-$(CH_2)_6$-NBn_2**

To a suspension of $LiAlH_4$ (0.36 g, 9.5 mmol) in freshly distilled unhydrous THF (6.6 mL), amide **9a or 9b or 9c** (1.9 mmol) was added portionwise under an Ar atmosphere. The resulting mixture was refluxed for 4 h (amide **9b**) or overnight (amides **9a** and **9c**), whereby TLC, using solvent system B

for development, indicated the end of the reaction. Following cooling at 0 °C, the excess of $LiAlH_4$ was carefully destroyed by the dropwise addition of a saturated aqueous solution of Na_2SO_4. The resulting white precipitate was filtered off under vacuo and washed on filter with distilled THF. The filtrate concentrated in vacuo to a small volume and diluted with EtOAc. The organic phase was washed twice with brine (saturated aqueous NaCl), dried over anhydrous Na_2SO_4 and finally evaporated to dryness under reduced pressure. The anticipated diamine derivatives were obtained pure after FCC using solvent system A as eluant.

S1.1.4.1. N^1,N^1-Dibenzyl-N^3-trityl-1,3-propanediamine [TrtNH-$(CH_2)_3$-NBn_2]

White solid; Yield: 60%; M.p. 84.9-87.2 °C; R*f* (A): 0.23.
ATR-IR (cm^{-1}): 3302, 1598, 773, 747, 735, 694.
MS (ESI, 30 eV): *m/z* 535.51 [M+K], 519.52 [M+Na], 497.56 [M+1], 243.57 [Trt].
^{1}H-NMR (400.13, $CDCl_3$, δ): 7.42 (6H, d, *J* = 7.6 Hz), 7.31-7.18 (19H, m), 3.48 (4H, br. s), 2.47 (2H, unresolv. t), 2.17 (2H, t, *J* = 6.4 Hz), 1.74 (2H, unersolv. quint.), 1.61 (1H, br. s) ppm.
^{13}C-NMR (100.61, $CDCl_3$, δ): 146.2 (3C), 139.6, 128.8 (2C), 128.6 (6C), 128.1 (2C), 127.9 (2C), 127.7 (6C), 127.2, 126.7 (2C), 126.1 (5C), 70.9, 58.2 (2C), 51.0, 41.5, 27.8 ppm.

S1.1.4.2. N^1,N^1-Dibenzyl-N^4-trityl-1,4-butanediamine [TrtNH-$(CH_2)_4$-NBn_2]

Pale yellow oil; Yield: 80%; R*f* (A): 0.2.
ATR-IR (cm^{-1}): 3300, 1596, 747, 694.
MS (ESI, 30 eV): *m/z* 549.51 [M+K], 533.61 [M+Na], 511.58 [M+H], 243.50 [Trt].
^{1}H-NMR (400.13, $CDCl_3$, δ): 7.47 (6H, d, *J* = 7.6 Hz), 7.38-7.18 (19H, three m), 3.56 (4H, br. s), 2.41(2H, unresolv. t), 2.08 (2H, t, *J* = 6.8 Hz), 1.57-1.46 (5H, two m) ppm.
^{13}C-NMR (100.61, $CDCl_3$, δ): 146.3 (3C), 139.9 (2C), 128.7 (2C), 128.6 (6C), 128.1 (2C), 127.9 (2C), 127.7 (6C), 127.2, 126.7 (2C), 126.1 (5C), 70.8, 58.2 (2C), 53.3, 43.4, 28.4, 24.8 ppm.

S1.1.4.3. N^1,N^1-Dibenzyl-N^6-trityl-1,6-hexanediamine [TrtNH-$(CH_2)_6$-NBn_2]

White solid; Yield: 80%; M.p. 98-100 °C; R*f* (B): 0. 32.
ATR-IR (cm^{-1}): 3301, 1595, 739, 696.
MS (ESI, 30 eV): *m/z* 577.42 [M+K], 561.46 [M+Na], 539.55 [M+H], 243.64 [Trt].
^{1}H-NMR (400.13, $CDCl_3$, δ): 7.48 (6H, m), 7.38-7.14 (19H, three m), 3.55 (4H, br. s), 2.39 (2H, unresolv. t), 2.09 (2H, t, *J* = 6.8 Hz), 1.51-1.43 (5H, two m), 1.22 (4H, m) ppm.
^{13}C-NMR (100.61, $CDCl_3$, δ): 146.3 (3C), 140.0 (2C), 128.7 (4C), 128.6 (6C), 128.1 (4C), 127.7 (6C), 126.7 (2C), 126.1 (3C), 70.8, 58.2 (2C), 53.2, 43.5, 30.8, 27.1 (2C), 26.9 ppm.

S1.1.5. General procedure for the preparation of the N^1,N^1-dibenzylated diamines as their corresponding bistrifluoroacetate salts 10

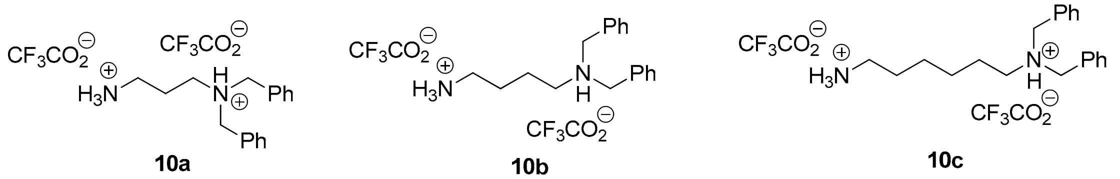

10a 10b 10c

To an ice-cold solution of the diamine derivative TrtNH-$(CH_2)_n$-NBn_2 (0.57 mmol) in CH_2Cl_2 (1.7 mL), were added sequentially TES (0.18 mL, 1.14 mmol) and TFA (0.28 mL, 3.3 mmol). The resulting solution was kept at that temperature for 15 min and then at ambient temperature for 1.5 h. The progress of the reaction was followed by TLC using the solvent system C (initially) and then the solvent systems J and L as eluant. Following completion of the reaction, Et_2O and then hexane were

added to the reaction mixture and the desired product was obtained either as a solid (**10b**) after filtration or oil (**10a** or **10c**) after decanting of the supernatant solvents. The products were dried overnight, under vacuo, over P_2O_5 and used as such into the following coupling reaction.

S1.1.5.1. *N*[1],*N*[1]-Dibenzyl-1,3-propanediamine bistrifluoroacetate (10a)

Pale yellow oil; Yield: 85%; R*f* (J): 0.47.
MS (ESI, 30 eV): *m*/*z* 255.51 [M+H], 198.51 [Bn_2NH+H].

S1.1.5.2. *N*[1],*N*[1]-Dibenzyl-1,4-butanediamine bistrifluoroacetate (10b)

White solid; Yield: 95%; M.p. 162.7-163.7 °C; R*f* (L): 0. 17.
MS (ESI, 30 eV): *m*/*z* 269.63 [M+H], 198.57 [Bn_2NH+H].

S1.1.5.3. *N*[1],*N*[1]-Dibenzyl-1,6-hexanediamine bistrifluoroacetate (10c)

Pale yellow oil; Yield: 87%; R*f* (J): 0.51.
MS (ESI, 30 eV)**:** *m*/*z* 619.52 [2M+Na], 198.51 [Bn_2NH+H].

S1.1.6. Synthesis of *N*[1],*N*[1],*N*[8],*N*[8]-tetrabenzyl-4-aza-5-oxaoctadiamide (11)

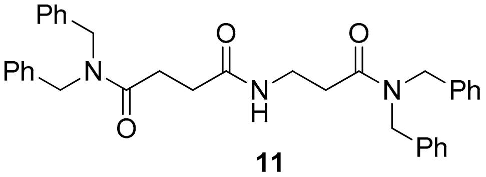

11

To an ice-cold solution of amide **9a** (0.28 g, 0.55 mmol) in CH_2Cl_2 (2.8 mL), were added sequentially TES (0.11 mL, 0.67 mmol) and TFA (0.57 mL, 6.8 mmol). The resulting solution was kept at that temperature for 15 min and then at ambient temperature for 30 min when the reaction was completed (TLC, solvent system D as eluant). Et_2O and then hexane were added to the reaction mixture and the desired product was obtained as an oil, after decanting the supernatant solvents and drying overnight, under vacuo, over P_2O_5. The product (trifluoroacetate salt) was used as such into the synthesis of triamide **11**, as described below.
Pale yellow oil; Yield: 95%; R*f* (I): 0.28.
MS (ESI, 30 eV)**:** *m*/*z* 269.70 [M+H], 198.70 [Bn_2NH+H].

To a solution of the thus above obtained trifluoroacetate salt in DMF (1.8 mL), succinic anhydride (55 mg, 0.55 mmol) was added at room temperature. The resulting solution was cooled to 0 °C and DIEA (0.19 mL, 1.1 mmol) was added. When the acylation reaction was found complete (TLC, solvent system M as eluant), HBTU (0.27 g, 0.71 mmol) and Bn_2NH (0.12 mL, 0.63 mmol) was added sequentially. The pH of the reaction was readjusted to 8 by adding additional DIEA (0.1 mL, 0.55 mmol). After 3.5 h at ambient temperature, when the second acylation reaction was found complete (TLC), the reaction mixture was diluted with EtOAc and the organic phase was sequentially washed once with 5% aqueous $NaHCO_3$, twice with H_2O and once with brine. Drying (anhydrous Na_2SO_4) and evaporation of the solvent under reduced pressure gave an oily residue, from which pure triamide **11** was obtained by FCC and using solvent system G as eluant.

Pale yellow oil; Yield: 90%; R*f* (G): 0.16.
ATR-IR (cm^{-1}): 3200, 1635, 730, 695.
MS (ESI, 30 eV): *m*/*z* 1117.79 [2M+Na], 586.33 [M+K], 570.43 [M+Na], 548.46 [M+H].

^{1}H-NMR (400.13, $CDCl_3$, δ): 7.38-7.10 (20H, three m), 6.74 (1H, unresolv. t), 4.60 (4H, two overlapping s), 4.49 (2H, s) και 4.40 (2H, s), 3.61 (2H, q, J = 5.6 Hz), 2.78 (2H, t, J = 6.8 Hz), 2.64 (2H, t, J = 5.6 Hz), 2.59 (2H, t, J = 6.8 Hz) ppm.
^{13}C-NMR (150.9, $CDCl_3$, δ): 172.6, 172.4, 172.2, 137.0, 136.9, 136.2 (2C), 136.0 (2C), 129.0 (2C), 128.9 (2C), 128.6 (2C), 128.1 (4C), 127.7, 127.6, 127.5, 127.4, 126.5 (2C), 126.4 (2C), 50.0, 49.9, 48.4, 48.3, 35.5, 32.9, 31.4, 28.5 ppm.

S1.1.7. Synthesis of N^1,N^1,N^8,N^8-tetrabenzylspermidine (12)

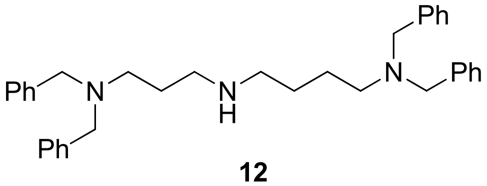

12

To a suspension of $LiAlH_4$ (0.21 g, 5.48 mmol) in freshly distilled dry THF (2.9 mL), triamide **11** (1.9 mmol) was added portionwise under an Ar atmosphere. The resulting mixture was refluxed overnight (12 h), whereby TLC, using solvent system N for development, indicated the end of the reaction. Following cooling at 0 °C, the excess of $LiAlH_4$ was carefully destroyed by the dropwise addition of a saturated aqueous solution of Na_2SO_4. The resulting white precipitate was filtered off under vacuo and washed on filter with distilled THF. The filtrate concentrated in vacuo to a small volume and diluted with EtOAc. The organic phase was washed twice with brine (saturated aqueous NaCl), dried over anhydrous Na_2SO_4 and finally evaporated to dryness under reduced pressure. The anticipated spermidine derivative **12** was obtained pure after FCC using solvent system K as eluant. Yellow oil; Yield: 90%; R*f* (K): 0.14.
ATR-IR (cm^{-1}): 3085, 3058, 3025, 2932, 1496, 1450, 1365, 1125, 1030, 727, 694.
MS (ESI, 30 eV): *m/z* 544.43 [M+K], 528.60 [M+Na], 506.63 [M+H].
^{1}H-NMR (600.13, $CDCl_3$, δ): 7.37-7.21 (20H, three m), 3.55 and 3.54 (8H, two overlapping br. s), 2.62 (2H, t, J = 6.6 Hz), 2.49 (2H, t, J = 6.6 Hz), 2.45 and 2.42 (4H, two overlapping t, J = 6.6 Hz), 1.81 (2H, quintet, J = 6.6 Hz), 1.56-1.49 (5H, m) ppm.
^{13}C-NMR (150.9, $CDCl_3$, δ): 139.7 (2C), 139.1 (2C), 129.0 (2C), 128.8 (2C), 128.3 (2C), 128.2 (2C), 127.1 (2C), 126.8 (2C), 58.3 (4C), 52.8, 51.4, 48.7, 47.7, 26.0, 25.1, 24.7 ppm.

S1.1.8. General procedure for the synthesis of the PA-CAM conjugates 4-7

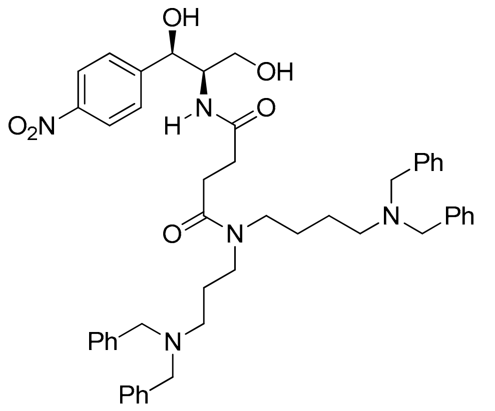

4

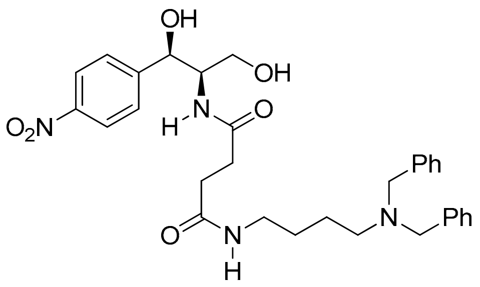

5

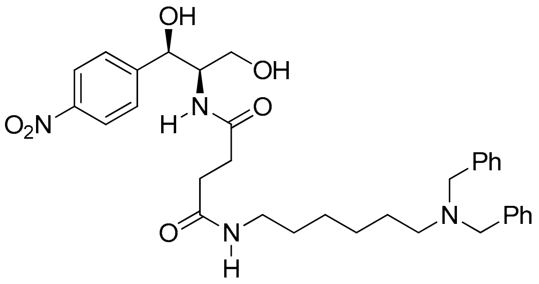

6

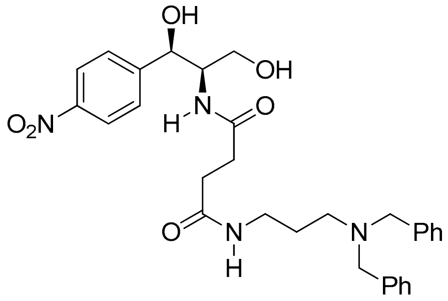

7

To a solution of CLB (55 mg, 0.26 mmol) in dry DMF (1 mL), succinic anhydride (26 mg, 0.26 mmol) was added and the reaction mixture was stirred at ambient temperature for 1.5 h, whereby the acylation reaction was found complete (TLC, solvent system J). The reaction mixture was then cooled to 0 °C and polyamine derivatives **10a** or **10b** or **10c** or **12** (0.30 mmol) added, followed by the sequential addition of HBTU (129 mg, 0.34 mmol) and DIEA (0.21 mL, 1.2 mmol). The resulting reaction mixture was stirred at ambient temperature for additional 2 h and then diluted with EtOAc. The organic phase was washed sequentially twice with 5% aqueous $NaHCO_3$, twice with H_2O and once with brine, and then dried over anhydrous Na_2SO_4. The solvent evaporated under reduced pressure and the anticipated PA-CAM conjugates were obtained pure by FCC using as eluant the solvent system I for conjugates **5**-**7** and H for conjugate **4**.

S1.1.8.1 Conjugate 4

Yellow oil; Yield: 60%; R*f* (H): 0.30.
ATR-IR (cm^{-1}): 3352, 1618, 727, 694.
MS (ESI, 30 eV): *m*/*z* 822.52 [M+Na], 800.70 [M+H].
^{1}H-NMR (400.13, $CDCl_3$, *δ*): 8.16 (2H, d, *J* = 8.4 Hz), 7.55 (2H, unresolv. d), 7.38-7.23 (20H, two m), 6.72 (1H, d, *J* = 6.4 Hz), 5.16 (1H, d, *J* = 4.0 Hz), 3.95-3.85 (2H, m), 3.76-3.70 (1H, m), 3.56 (8H, br. s), 3.18-2.95 (4H, m), 2.67-2.56, 2.44-2.35 and 2.31-2.26 (8H, three m), 1.68-1.64 (2H, m), 1.44-1.37 (4H, m) ppm.
^{13}C-NMR (100.61, $CDCl_3$, *δ*): 174.2, 171.8, 148.9, 147.2, 139.2 (2C), 128.9 (2C), 128.8 (4C), 128.7 (4C), 128.3 (4C), 128.2 (4C), 127.1 (2C), 127.0 (2C), 126.9 (2C), 123.4 (2C), 73.5, 63.1, 58.8, 58.6, 58.3, 58.1, 57.2, 52.8, 50.9, 47.9, 46.1, 31.7, 29.1, 26.2, 25.4, 24.4 ppm.

S1.1.8.2. Conjugate 5

Yellow oil; Yield: 60%; R*f* (I): 0.27.
ATR-IR (cm^{-1}): 3290, 1645, 740, 670.
MS (ESI, 30 eV): *m/z* 601.58 [M+K], 585.53 [M+Na], 563.64 [M+H].
^{1}H-NMR (400.13, $CDCl_3$, *δ*): 8.15 (2H, d, *J* = 8.0 Hz), 7.54 (2H, d, *J* = 8.0 Hz), 7.40-7.28 (10H, m), 7.0 (1H, unresolv. d), 6.15 (1H, unresolv. t), 5.16 (1H, unresolv. d), 4.00 (1H, unresolv. m), 3.86-3.81 (1H, m), 3.74 (5H, br. s), 3.14-3.10 (2H, m), 2.56-2.34 (6H, m), 1.61 (2H, unresolv. q, H-22), 1.42 (2H, unresolv. quintet) ppm.
^{13}C-NMR (150.9, $CDCl_3$, *δ*): 171.8, 171.3, 148.9, 147.2, 129.5 (2C), 128.6 (8C), 127.9 (2C), 126.9 (2C), 123.4 (2C), 73.4, 63.2, 57.9 (2C), 56.9, 52.5, 38.9, 31.9, 31.7, 26.9, 23.5 ppm.

S1.1.8.3. Conjugate 6

Yellow oil; Yield: 62%; R*f* (I): 0.33.
ATR-IR (cm^{-1}): 3310, 1640, 739, 696.
MS (ESI, 30 eV): *m/z* 613.35 [M+Na], 591.51 [M+H].
^{1}H-NMR (400.13, $CDCl_3$, *δ*): 8.15 (2H, d, *J* = 8.0 Hz), 7.57 (2H, d, *J* = 8.4 Hz), 7.39-7.25 (10H, m), 6.99 (1H, unresolv. d), 6.08 (1H, unresolv. t), 5.18 (1H, unresolv. d), 4.09 (1H, unresolv. dd), 3.84 (1H, unresolv. dd) 3.73 (1H, unresolv. dd), 3.65 (4H, br. s), 3.11 (2H, m), 2.49 -2.36 (6H, two m), 1.55 (2H, quint., *J* = 8.0 Hz), 1.40 (2H, quint., *J* =8.0 Hz), 1.29-1.18 (4H, m) ppm.
^{13}C-NMR (150.9, $CDCl_3$, *δ*): 173.3, 172.4, 149.1, 147.2, 129.1 (4C), 128.3 (6C), 127.3 (2C), 126.9 (2C), 123.9 (2C), 72.9, 62.9, 58.0 (2C), 56.5, 52.9, 39.6, 31.6, 31.5, 29.2, 26.6, 26.4, 26.3 ppm.

S1.1.8.4. Conjugate 7

Yellow oil; Yield: 73%; R*f* (I): 0.16.
ATR-IR (cm^{-1}): 3310 (broad), 1620, 738, 697.
MS (ESI, 30 eV): *m/z* 571.62 [M+Na], 549.58 [M+H].
^{1}H-NMR (400.13, $CDCl_3$, *δ*): 8.17 (2H, d, *J* = 8.4 Hz), 7.57 (2H, d, *J* = 8.8 Hz), 7.37-7.27 (10H, m), 6.91 (1H, d, *J* = 6.0 Hz), 6.0 (1H, br. s), 5.17 (1H, d, *J* = 3.2 Hz), 4.03 (1H, m), 3.87 (1H, dd, *J* = 4.0 and 11.4 Hz), 3.76 (1H, dd, *J* = 3.6 and 11.4 Hz) 3.56 (4H, br. s), 3.20 (2H, q, *J* = 6.0 Hz), 2.49 (2H, unresolv. t), 2.37-2.21 (3H, m), 2.14-2.08 (1H, m), 1.63 (2H, quint,, *J* = 5.6 Hz) ppm.
^{13}C-NMR (150.9, $CDCl_3$, *δ*): 173.4, 172.3, 149.0, 147.2, 129.2 (4C), 128.5 (6C), 127.4 (2C), 126.9 (2C), 123.4 (2C), 73.3, 63.1, 58.4 (2C), 56.7, 50.2, 37.6, 31.6, 31.2,
25.6 ppm.

S2. Results section

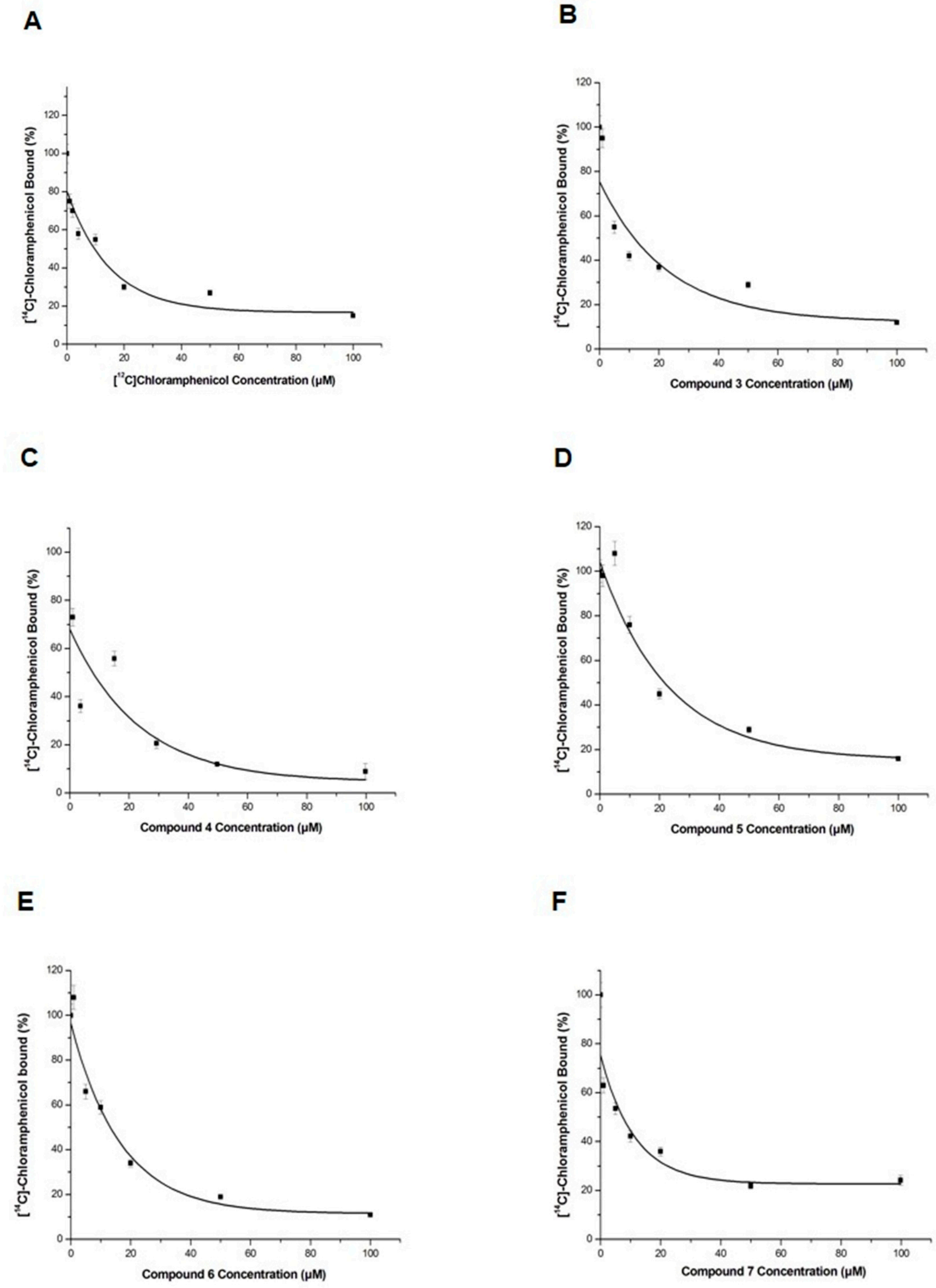

Figure S1. Competitive binding of [^{14}C]-CAM with A) non-radioactive labeled CAM, B) Compound **3**, C) Compound **4**, D) Compound **5**, E) Compound **6**, F) Compound 7. The diagrams represent the percentage of displacement of [^{14}C]-CAM using increasing concentrations of each compound.

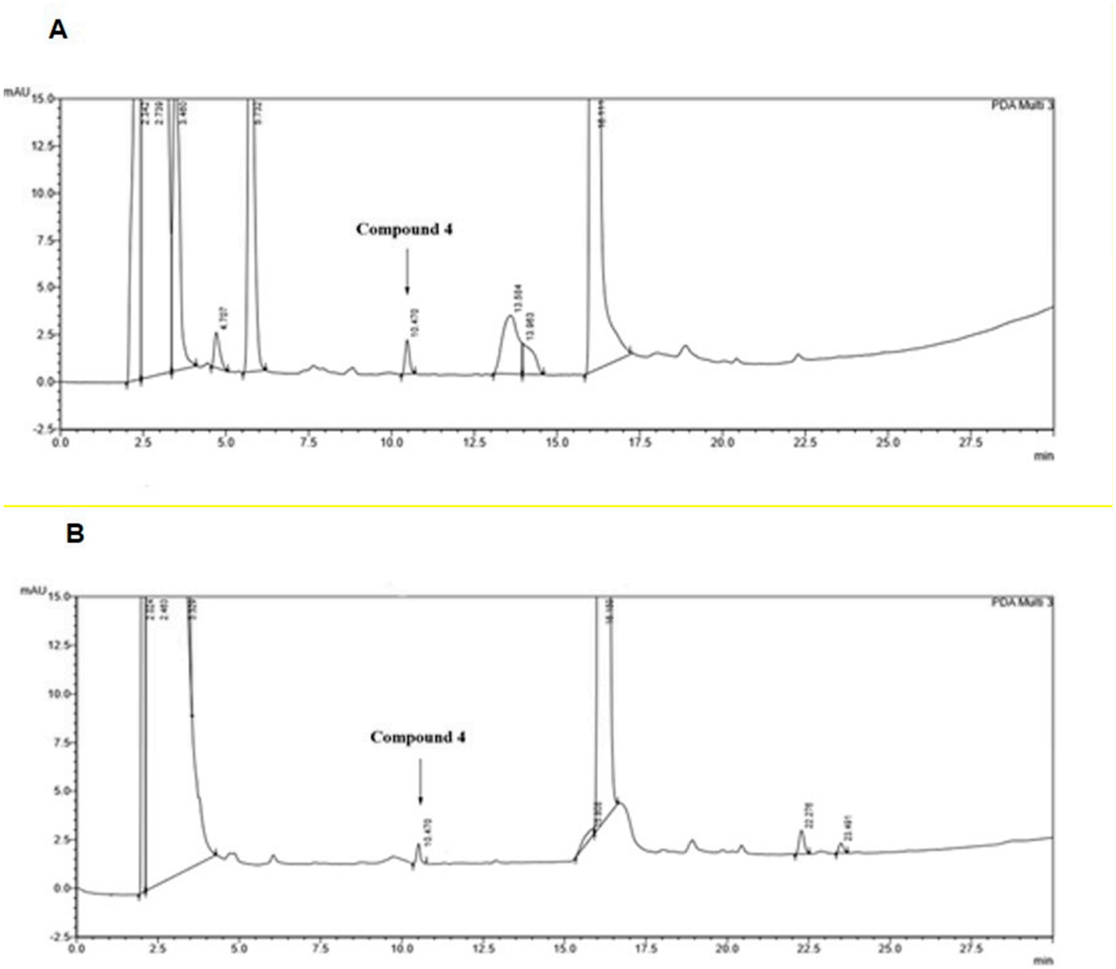

Figure S2. Representative chromatograms of RP-HPLC analysis for the intracellular concentration of compound **4**, when A) ZL34 and B) Met5A cells were challenged by this compound at concentrations of 60 μM. Optical absorbance of compound was measured at 275 nm.

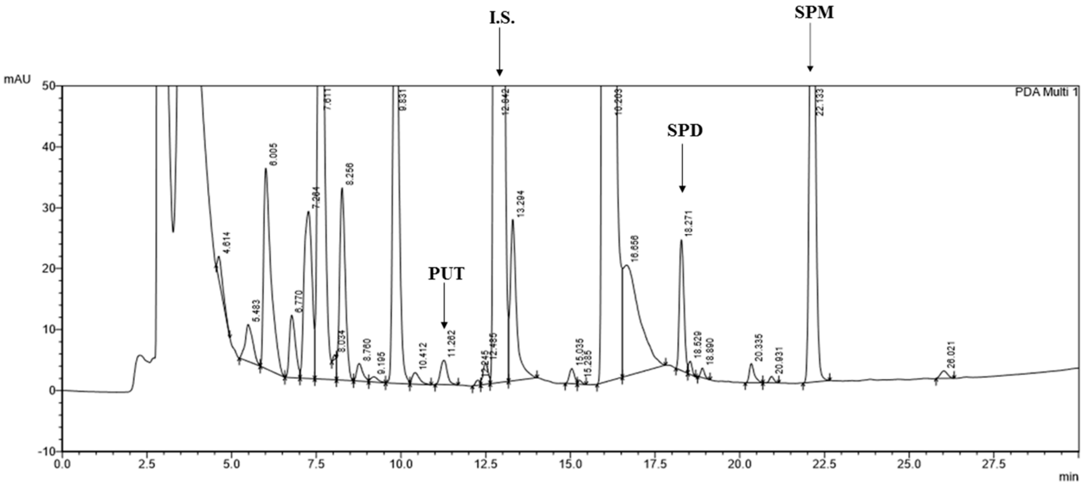

Figure S3. Representative chromatogram of RP-HPLC analysis of ZL34 cells in the absence of compounds tested. The peaks corresponding to dansylated polyamines are indicated by arrows. PUT, putrescine; SPD, spermidine; SPM, spermine; I.S., internal standard. Identification of the peak of each polyamine was made using three standard samples of known concentrations. Verification of the identity of each peak was done by comparing the UV spectrum.

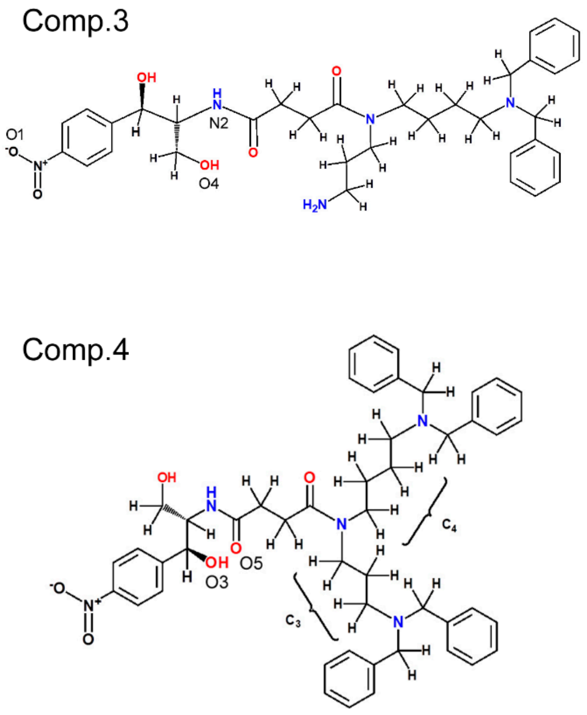

Figure S4. Atom numbering in the compound **3** and **4** models, given by Arguslab.

S3. Materials and Methods Section

Table S1: Intersystem Correspondence of Relevant Large Subunit Nucleotides. The matching of RNA nucleotides was based on data from Brown et al. [33].

***E. coli* 23S rRNA**	**Human mitochondrial 16S rRNA**
A2058	G2721
A2059	A2722
A2062	A2725
A2451	A2938
C2452	C2939
G2505	G2992
U2506	U2993
U2585	U3072
U2586	U3073
A2062	A3089
